# Dobutamine stress testing in patients with Fontan circulation augmented by biomechanical modeling

**DOI:** 10.1371/journal.pone.0229015

**Published:** 2020-02-21

**Authors:** Bram Ruijsink, Konrad Zugaj, James Wong, Kuberan Pushparajah, Tarique Hussain, Philippe Moireau, Reza Razavi, Dominique Chapelle, Radomír Chabiniok

**Affiliations:** 1 School of Biomedical Engineering & Imaging Sciences, St Thomas’ Hospital, King’s College London, London, England, United Kingdom; 2 Department of Pediatrics, UT Southwestern Medical Center, Dallas, TX, United States of America; 3 Inria Centre de Recherche Saclay-Ile-de-France, Palaiseau, France; 4 LMS, Ecole Polytechnique, CNRS, Institut Polytechnique de Paris, Paris, France; Temple University, UNITED STATES

## Abstract

Understanding (patho)physiological phenomena and mechanisms of failure in patients with Fontan circulation—a surgically established circulation for patients born with a functionally single ventricle—remains challenging due to the complex hemodynamics and high inter-patient variations in anatomy and function. In this work, we present a biomechanical model of the heart and circulation to augment the diagnostic evaluation of Fontan patients with early-stage heart failure. The proposed framework employs a reduced-order model of heart coupled with a simplified circulation including venous return, creating a closed-loop system. We deploy this framework to augment the information from data obtained during combined cardiac catheterization and magnetic resonance exams (XMR), performed at rest and during dobutamine stress in 9 children with Fontan circulation and 2 biventricular controls. We demonstrate that our modeling framework enables patient-specific investigation of myocardial stiffness, contractility at rest, contractile reserve during stress and changes in vascular resistance. Hereby, the model allows to identify key factors underlying the pathophysiological response to stress in these patients. In addition, the rapid personalization of the model to patient data and fast simulation of cardiac cycles make our framework directly applicable in a clinical workflow. We conclude that the proposed modeling framework is a valuable addition to the current clinical diagnostic XMR exam that helps to explain patient-specific stress hemodynamics and can identify potential mechanisms of failure in patients with Fontan circulation.

## Introduction

The Fontan circulation is a surgically created single-ventricular circulation for patients with complex congenital heart diseases in whom one of the ventricles is unable to support circulation [1]. In the Fontan circulation, the systemic venous return is rerouted to the lungs via a direct connection of venae cavae to the pulmonary arteries, the so-called total cavopulmonary connection (TCPC). As a result, the single existing ventricle acts as a pump for systemic and pulmonary circulations in series.

Patients with Fontan circulation experience a progressive decline in cardiac function over time and a high incidence of heart failure [2]. The mechanism of Fontan failure in individual patients is often multifactorial and can include: myocardial dysfunction; altered resistance and compliance in the pulmonary and/or systemic vascular beds; reduced systemic arterial oxygen content due to pathological shunting; or reduced systemic or pulmonary venous return with subsequent impaired filling of the ventricle. The main component of failure in an individual patient is hard to detect using routine clinical diagnostic examinations. For instance, conventional measures of cardiac function, such as ejection fraction (EF), do not represent the true underlying state of the single-ventricle due to abnormal loading conditions in the Fontan circulation [3, 4]. Simultaneous cardiovascular magnetic resonance imaging (CMR) and catheterization (XMR) exams at rest and during pharmacological stress are currently employed in several specialized congenital cardiac centers to investigate the causes of heart failure in Fontan patients [5]. These exams provide rich datasets of combined pressure and flow measurements. However, the complex physiology of Fontan circulation and large inter-individual variations make it difficult to obtain direct conclusions. Integrating all data obtained during the XMR stress exam into a single diagnostic framework can potentially improve the understanding of the pathophysiological mechanisms underlying Fontan failure in individual patients, and biophysical models interacting with the clinical data have a great potential in this respect.

### Modeling Fontan physiology

Biophysical modeling has advanced in recent years into clinical applications by assisting diagnosis [6, 7] or therapy planning [8–10]. As opposed to image or signal analysis, the physical and physiological principles of these models allow to estimate the biophysical properties of the heart and vascular system—quantities not directly measurable from the image and pressure data but accessible via constitutive relations and equilibrium equations (see recent reviews [11, 12] and references therein). Modeling techniques have already been applied to investigate Fontan physiology. Most works have so far focused on investigating flow characteristics in the TCPC and the effect of alternative surgical TCPC configurations on blood flow through the pulmonary system [13–16]. Fewer works have addressed modeling of the heart itself.

Implementing a full 3D biophysical model provides a valuable insight into the single-ventricular function. For instance, in [17] fluid-structure interaction has been used to investigate diastolic function of the single-ventricle. However, simulating a full cardiac cycle of the ventricle looped with vascular system under varying physiological conditions (e.g. during stress) using a 3D approach would lead to high computational expenses. While such computation power could be available in specialized biomedical research laboratories, they are currently not readily accessible in a routine clinical setting. Even if available, for example through cloud computing, personalization of complex 3D models requires expert input. These constraints limit their implementation directly into the clinical diagnostic workflow. Moreover, while they have significant added values in certain areas where spatial differences in myocardial function are relevant—e.g. to investigate local foci in arrythmogenic diseases or predict the effect of cardiac resynchronization therapy [8]—the assessment of global biophysical properties alone can already allow significant improvements in the diagnostic assessment of the cardiovascular mechanisms underlying early-stage failure in Fontan patients.

A number of groups have studied Fontan physiology using lumped-parameter models, in which the heart is typically represented by a varying elastance model based on experimental works of Suga et al. [18]. Closed-loop models of this type, based on population trends, have given an insight into the (patho)physiology of Fontan circulation [19, 20]. However, the limited amount of detailed patient-specific biophysical components reduces the predictions for individual patients. Even though patient-specific lumped-parameter models have been proposed [21–23], their personalization may be intricate, and predictive capabilities inferior compared to biophysical multiscale models due to their mostly phenomenological background.

Caruel et al. [24] have decreased the complexity of a multiscale biomechanical heart model [25, 26] by reducing the 3D anatomy of patients’ ventricles by a spherical symmetry approximation. While geometry and kinematics are thus reduced, all other biophysical properties correspond to the full 3D model. This model combines the advantage of physical and physiological backgrounds, as well as fast computation allowing a close-to-real time interaction with clinical data. Indeed, the physical character of the model allows to implement a sequential calibration procedure turning the model into a patient-specific regime, which can then augment the information obtained from the data, and perform it directly on site without a need of high-performance computing resources [10, 27].

### Aims

In this work, we aim to develop a patient-specific modeling framework to augment the diagnostic value of XMR stress exams in Fontan patients with early-stage heart failure. We employ the reduced-order cardiac model [24] with a closed-loop circulation [9, 28] to evaluate key clinical parameters of cardiovascular function—myocardial stiffness, myocardial contractility, contractile reserve and vascular resistance—in individual patients at rest and under pharmacological stress.

## Materials and methods

### XMR data acquisition

Nine patients with Fontan circulation (*FP#1-9*), who underwent an XMR dobutamine stress exam for progressive symptoms of exercise intolerance (early-stage heart failure), were included in this study. In all *FPs*, the underlying congenital heart defect was hypoplastic left heart syndrome. None of them had clinically significant aorto-pulmonary or veno-venous collaterals. Additionally, two “control cases” (*CC#1* and *#2*) with a biventricular heart and normal circulation were included. These control patients suffered from Allagile’s syndrome—billiary atresia—and had no clinically significant pulmonary artery stenoses. They underwent a clinical dobutamine stress XMR during work-up for liver transplantation.

**Fig 1:**
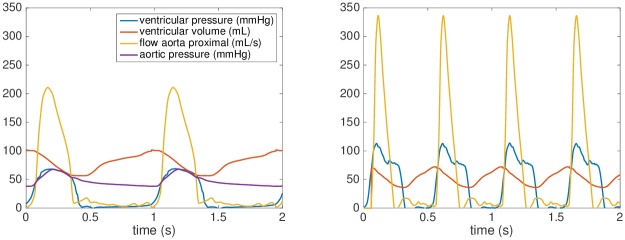
XMR data. Processed data of a selected Fontan patient acquired at rest (left) and during dobutamine stress (right).

All XMR exams were performed under general anesthesia. During XMR, pressure signals (aortic, ventricular, venae cavae and pulmonary capillary wedge pressures) were obtained at rest, and under continuous infusion of dobutamine 10 *μ*g/kg of body weight /min (*stress*). Simultaneously, cardiac volumes and 2D flows (ascending aorta, descending aorta, venae cavae, and branch pulmonary arteries) were acquired using CMR. The obtained CMR data were post-processed into 0D signals of time-vs-flow or time-vs-ventricular volume, see Fig 1. This study was performed under the ethical approval of our institutional ethics committee, London UK (Ethics Number 09H0804062), and the parents of all included patients gave written consent for participation in the study.

### Biomechanical model

**Fig 2:**
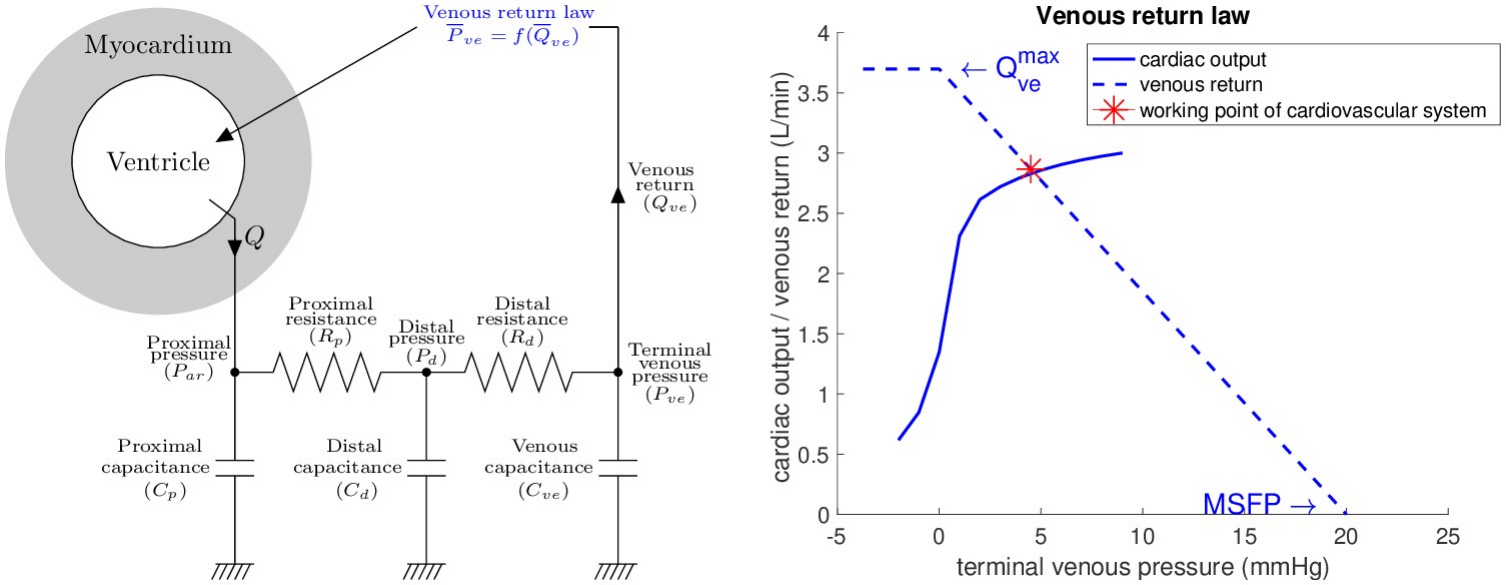
Scheme of the closed-loop heart-circulation model. The circuit is closed by linear venous return law ${\bar{P}}_{ve}=f\left({\bar{Q}}_{ve}\right)$ (blue dashed line in the right panel given by Eq (9)), which defines the venous pressure and preload of systemic ventricle according to the cardiac output (MSFP stands for mean systemic filling pressure and $Q_{ve}^{max}$ for maximum achievable venous flow).

The model used in the presented study includes the components of heart ventricle and circulation connected into a closed-loop system. The ventricle is modeled by using the multiscale biomechanical model described in detail in [25] and [26], of which the geometry and kinematics were reduced to a sphere [24], while all other biophysical properties correspond to the complex full 3D model. The ventricle is connected to circulation system represented by a Windkessel model. The circuit is closed by using a “Guytonian” venous return [9, 28]—i.e. the venous return is linearly proportional to the atrial pressure while assuming that at steady-state the venous return and cardiac output are in equilibrium. The principal unknowns of the model are the displacement of the “spherical ventricle” in the radial direction (capturing the variations of ventricular volume and systolic wall thickening during the cardiac cycle) and pressure in the ventricular cavity. The main ingredients of the model are shown in Fig 2 and described below.

The hyperelastic potential of the passive part of myocardium is in the form

$$\begin{array}{c} W_{e}=C_{0}e^{C_{1}{\left(J_{1}-3\right)}^{2}}+C_{2}e^{C_{3}{\left(J_{4}-1\right)}^{2}}, \end{array}$$ (1)

where *J*_1_ and *J*_4_ are reduced invariants of the right Cauchy-Green strain tensor $\underline{\underline{C}}$ [29], the latter corresponds to anisotropy in the orthoradial plane. The constitutive law is inspired by Holzapfel and Ogden [30] and was shown to provide a good balance between identifiability of parameters and model fidelity [31]. The material parameters *C*_0_, *C*_1_, *C*_2_ and *C*_3_ are pre-calibrated to obtain the end-diastolic pressure-volume relationship (EDPVR) according to experimental data [32].

The active component of the heart is modeled by a system of ordinary differential equations representing chemically controlled actin-myosin interactions [26]—formation of cross-bridges—generating active stress *τ*_*c*_ and active stiffness *k*_*c*_ in sarcomeres with extension $e_{fib}=\frac{L}{L_{0}}$ (where *L*_0_ and *L* represent the reference and actual sarcomere lengths, respectively):

$$\begin{array}{c} \dot{k_{c}}=-(|u|+\alpha |{\dot{e}}_{fib}\left|\right)k_{c}+n_{0}k_{0}{\left|u\right|}_{+},\\ \dot{\tau_{c}}=-(|u|+\alpha |{\dot{e}}_{fib}\left|\right)\tau_{c}+n_{0}\sigma_{0}{\left|u\right|}_{+}+k_{c}{\dot{e}}_{fib}. \end{array}$$ (2)

**Fig 3:**
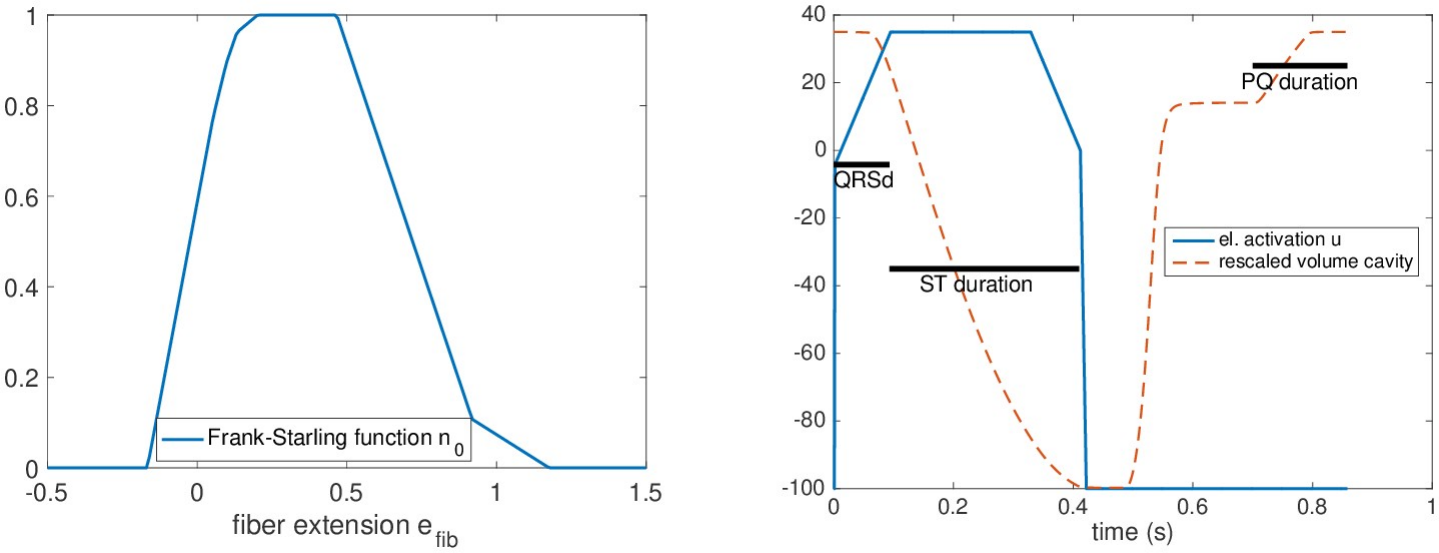
Frank-Starling mechanism and electrical activation Left: Maximum effect of the Frank-Starling mechanism (*n*_0_(e_*fib*_) = 1) at optimal fiber extension. Right: Profile of the electrical activation function *u* with a rescaled ventricular volume plot. The measured durations of QRS, PQ and ST segments of ECG at rest are used to impose timings for the electrical activation function and for the atrial contraction.

The asymptotic active stress *σ*_0_ and stiffness *k*_0_, generated by the sarcomere, are directly related to myocardial contractility, while taking into account the effect of actin-myosin overlap using a Frank-Starling law function *n*_0_(*e*_*fib*_) ∈ [0, 1] (adjusted using the experimental data from [33] and shown in Fig 3). The activation of the sarcomeres is modeled using an activation function *u*, which is positive when the tissue is electrically activated with the maximum value of 35 *s*^−1^ given by the rate of active stress generation [33]; |*u*|_+_ is defined as max(*u*, 0). The parameter *α* denotes the cross-bridge destruction rate due to rapid length changes [26].

The peripheral circulation, receiving flow *Q* from the heart, is modeled using a 2-stage Windkessel model [34]. The proximal part of the system (aorta and its first-order branches) is represented by a proximal resistance and capacitance *R*_*p*_, *C*_*p*_. The rest of the circulation, either the combined systemic and pulmonary beds (for Fontan patients) or solely the systemic bed (for biventricular patients), is represented by the distal resistance and capacitance (*R*_*d*_ and *C*_*d*_). The whole Windkessel system reads:

$$\begin{array}{c} C_{p}{\dot{P}}_{ar}+\left(P_{ar}-P_{d}\right)/R_{p}=Q,\\ C_{d}{\dot{P}}_{d}+\left(P_{d}-P_{ar}\right)/R_{p}=\left(P_{ve}-P_{d}\right)/R_{d}. \end{array}$$ (3)

The final modeling ingredient—the venous system—provides the preload to the systemic ventricle and closes the cardiovascular circuit. We employ a phenomenological observation of linear dependency of the venous return on atrial pressure substantiated by the experiments [28]. We will derive the venous return *Q*_*ve*_ from the blood conservation principle (see [9])

$$\begin{array}{c} P_{ve}C_{ve}+P_{d}C_{d}=V_{eff}, \end{array}$$ (4)

where *P*_*ve*_ represents the terminal venous pressure before entering the heart ventricle (we assume this pressure being the atrial pressure prior to the contraction of atria); *P*_*d*_ is the pressure associated to the distal systemic circulation; *C*_*ve*_ and *C*_*d*_ are the corresponding capacitances in the vascular system; and *V*_*eff*_ represents the amount of blood which is directly available for exchange between arterial and venous system.

Assuming steady-state situation in the terminal part of circulation, the venous flow can be written as

$$\begin{array}{c} Q_{ve}=\left(P_{d}-P_{ve}\right)/R_{d}. \end{array}$$ (5)

After substituting (5) into (4) we obtain

$$\begin{array}{c} Q_{ve}=\frac{V_{eff}}{R_{d}C_{d}}-(1+\frac{C_{ve}}{C_{d}})\frac{P_{ve}}{R_{d}}, \end{array}$$ (6)

leading to a linear relationship between the venous pressure *P*_*ve*_ and venous flow *Q*_*ve*_

$$\begin{array}{c} P_{ve}=\frac{V_{eff}}{C_{d}+C_{ve}}-Q_{ve}\frac{R_{d}C_{d}}{C_{d}+C_{ve}}=f\left(Q_{ve}\right). \end{array}$$ (7)

In stationary state, the venous return *Q*_*ve*_ must be in equilibrium with the cardiac output *Q*. The time integration of the simulated cardiac output *Q* over the reading frame Δ*t* of 6 cardiac cycles (with being *T*_0_ representing the cycle duration) will give

$$\begin{array}{c} {\bar{Q}}_{ve}\left(t\right)=\frac{\int_{t-\Delta t}^{t}e^{-\left(t-\tau \right)/T_{0}}Qd\tau }{\int_{t-\Delta t}^{t}e^{-\left(t-\tau \right)/T_{0}}d\tau }. \end{array}$$ (8)

We associate the low amplitude flow waveform ${\bar{Q}}_{ve}(t)$ (obtained by postprocessing the simulated cardiac output *Q*) with the terminal venous flow *Q*_*ve*_. Therefore, we rewrite Eq (7) into the form

$${\bar{P}}_{ve}=\frac{V_{eff}}{C_{d}+C_{ve}}-{\bar{Q}}_{ve}\frac{R_{d}C_{d}}{C_{d}+C_{ve}}=f({\bar{Q}}_{ve}).$$ (9)

The intersect of the linear venous law (9) with the x-axis represents the mean systemic filling pressure (MSFP)—the hypothetical pressure at zero flow (see Fig 2, right). The maximum venous return flow $Q_{ve}^{max}$ is reached at terminal venous pressure of 0 mmHg, with no further increase at negative pressures due to an assumed collapse of caval veins [28].

After adding the contribution of atrial contraction $P_{at}^{ramp}$ obtained from the data at rest, Eq (9) closes the loop by providing the preload pressure of the ventricle $P_{preload}={\bar{P}}_{ve}+P_{at}^{ramp}$.

### Model calibration

For each patient, the biomechanical model is calibrated using the measured data following a sequential protocol that results in a unique set of parameters. The points 1.–5. below describe the step-by-step calibration of patient-specific models at rest, and point 6. the model adjustment during dobutamine stress.

**Fig 4:**
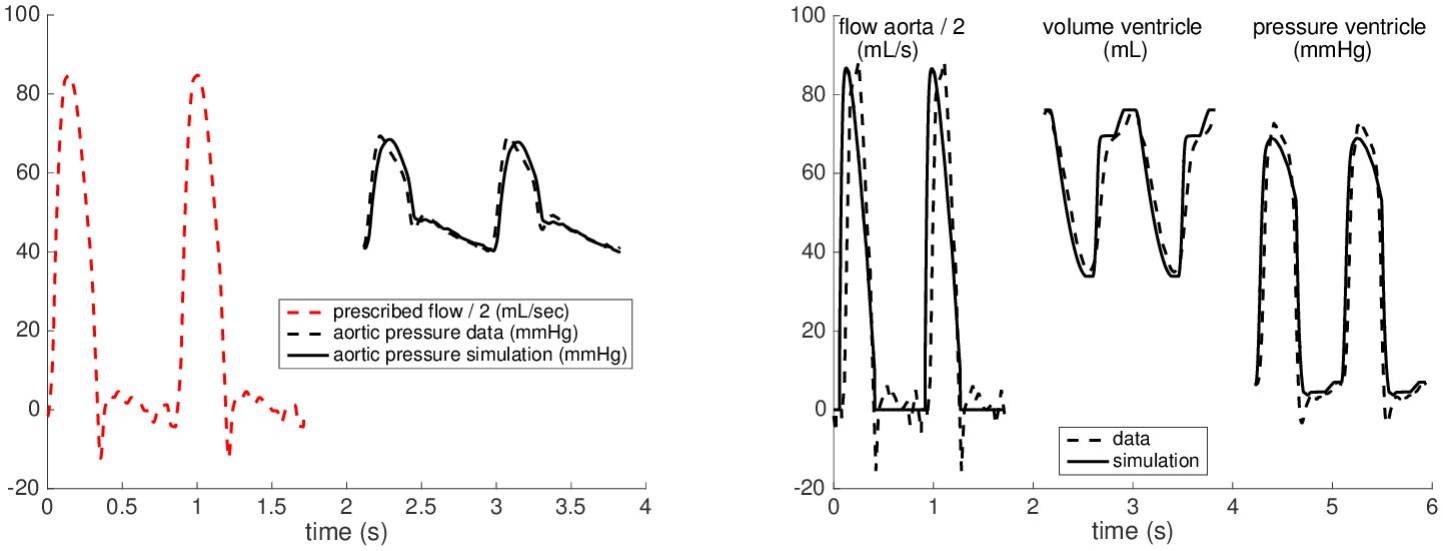
Model calibrations. Calibration of the Windkessel model (left), and of the heart model with prescribed level of preload (right) at rest. For visualization purposes, the aortic flow was downscaled by a factor of 2.

1 *Geometry of the ventricle*: The geometrical relations in the model of the ventricle (ventricular volume and wall thickness) are prescribed using the end-diastolic volume (EDV) and myocardial mass obtained from CMR. The ventricular volume at the reference (stress-free) configuration *V*_0_ is assumed to correspond to 50% of EDV at rest, which is close to the experimental assessments by Klotz et al. [32].
2 *Windkessel calibration*: The Windkessel model is decoupled from the heart and the aortic flow is imposed into the Windkessel system. Subsequently, the resistances and capacitances are adjusted to match the aortic pressure waveform in the data. The distal properties, *R*_*d*_ and *C*_*d*_, are calibrated to obtain the height of the dicrotic notch and the time constant of the diastolic pressure decay, while the proximal resistance *R*_*p*_ is tuned to obtain the peak systolic pressure. The proximal capacitance *C*_*p*_ is set to a low value targeting the time constant of the fast-dynamic proximal Windkessel system being *R*_*p*_ ⋅ *C*_*p*_ = 0.005 seconds. Fig 4 (left) shows the result of calibration in a Fontan patient.
3 *Calibration of passive properties*: The passive tissue properties of the myocardium—the hyperelastic potential Eq (1)—are adjusted using the measured ventricular end-diastolic pressure (EDP) and EDV. After imposing EDP, the linear stiffness coefficients *C*_0_ and *C*_2_ are multiplied by a “relative passive stiffness parameter” to obtain the measured EDV. The use of the relative stiffness parameter facilitates comparison of stiffness values between patients.
4 *Calibration of active properties*: The calibration of the active component of the ventricular myocardium, given by Eq (2), consists of the adjustment of electrical activation function *u* and of myocardial contractility *σ*_0_. In this step, the heart model is connected to the calibrated Windkessel component and the measured EDP is imposed as preload. The profile of the function *u* and the atrio-ventricular activation delay are calibrated based on QRS, ST and PQ durations measured from the ECG at rest, see Fig 3 (right panel). After obtaining the personalized activation function, contractility *σ*_0_ is adjusted so that the simulation reaches the stroke volume (SV) and aortic flow observed in the data. Modeling the active atrial contraction is limited to prescribing a pressure increase $P_{at}^{ramp}$ corresponding to the measurement at rest with the timing corresponding to the PQ interval in the measured ECG. Fig 4 (right panel) demonstrates the results of calibration steps 1.-4. in a representative patient.
5 *Calibration of venous return*: The last step of the calibration of the closed-loop heart-circulation model at rest consists of identifying *V*_*eff*_ and *C*_*ve*_ in the venous return law (9). We parameterize the linear relationship using the working point of the heart at rest (intersection of the measured preload and cardiac output) marked by red star in Fig 2, and using the assumption of MSFP being 20 mmHg, which is in line with the measurements by Macé et al. [35] performed on a group of animals (pigs) with created Fontan circulations.
6 *Adaptation of the model to stress*: After calibrating the models at rest, the effects of dobutamine stress are introduced into the model. As for the resistance of the vascular system, the aortic pressure measurement was not available during stress. Since no aortic stenosis was observed in our group of patients, we directly associated the maximum aortic pressure with the measured peak ventricular pressure and used it to adjust the Windkessel model. We assumed that both the proximal and distal resistances will be modified by the same multiplication factor *DOB*_*WK*_, while the time constants of the system—i.e. *C*_*p*_ ⋅ *R*_*p*_ and *C*_*d*_ ⋅ *R*_*d*_—will remain unchanged. Therefore,
  $$\begin{array}{c} R_{p/d}^{stress}=R_{p/d}^{rest}\cdot DOB_{WK},\\ C_{p/d/ve}^{stress}=C_{p/d/ve}^{rest}/DOB_{WK}. \end{array}$$ (10)
  The chronotropic effect is accounted for by adjusting the cardiac cycle duration to the measured heart rate (HR). No reliable ECG was available during the dobutamine stress due to CMR-incompatibility of the ECG device. We assume that PQ and QRS durations do not change significantly during stress, while the ST interval is likely to shorten [36]. We therefore modified the ST interval to match the shortening of the measured ventricular pressure wave.
  Then, we adjusted the level of contractility to obtain the end-systolic volume (ESV) as in the data. Finally, if the EDV simulated by the closed-loop model was inconsistent with the data, the effective volume *V*_*eff*_—which directly controls the maximum venous return $Q_{ve}^{max}$ (we recall Eq (6))—was adjusted to account for the adaptation of the venous return at stress.

## Results

### Modeling outcomes at rest

**Fig 5:**
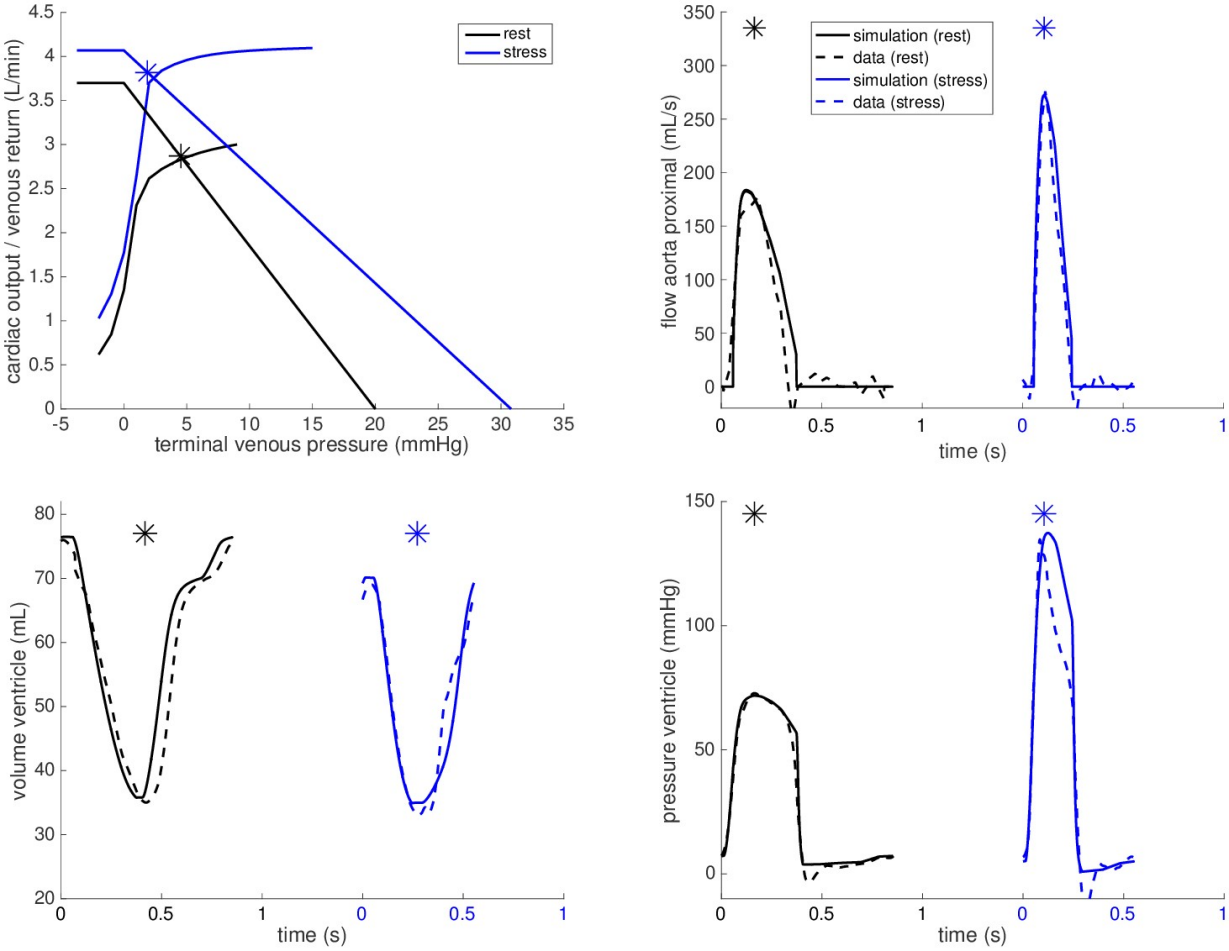
Example simulation for Fontan patient. Simulations for *FP#1* at rest (black) and during dobutamine stress (blue).

**Fig 6:**
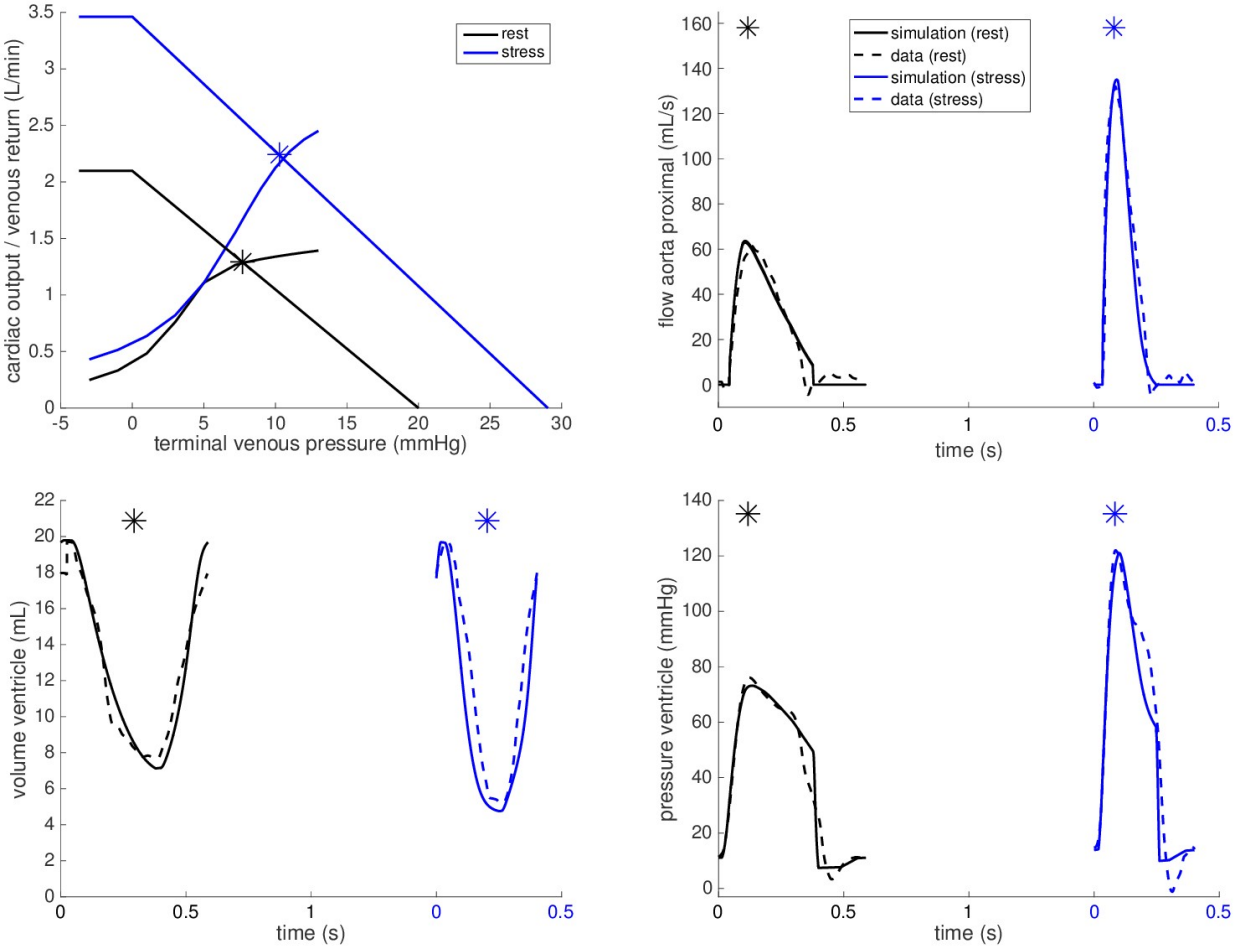
Example simulation for patient with biventricular heart. Simulations for *CC#1* at rest (black) and during dobutamine stress (blue). Note the significant upward shift of the venous return curve that improves the ability to augment CO during stress.

By following the calibration steps 1.-5. described in the Model calibration section, we set up the models at rest for all cases. The black plots in Figs 5 and 6 illustrate the calibrations of the closed-loop heart-circulation models at rest with respect to the measured data for a selected *FP* and *CC* (see Appendix for other subjects).

**Fig 7:**
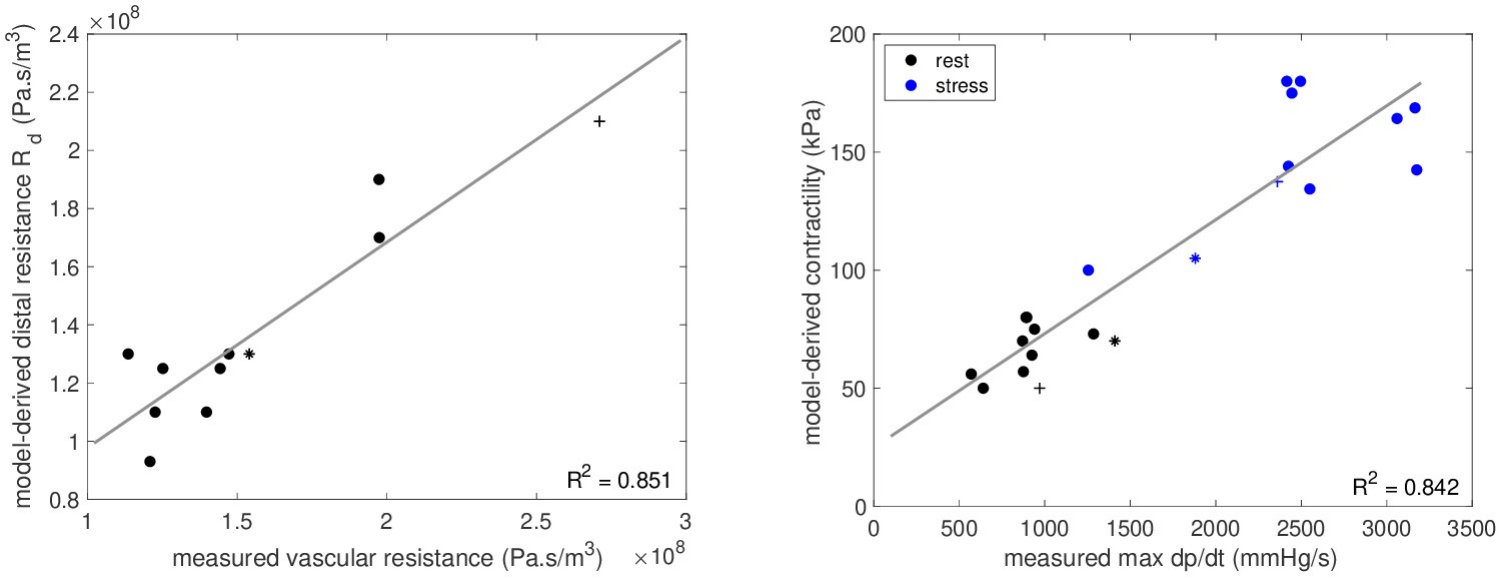
Model estimated quantities vs measurements. Left: Model-derived distal Windkessel resistance (*R*_*d*_) versus measured vascular resistance at rest. Right: Model estimated contractility versus measured ventricular max dp/dt. Markers • denote *FPs*, + denote *CC#1* and * denote *CC#2*.

Clinical measures for all included patients are shown in Table 1, and the parameters derived by the patient-specific models are listed in Table 2. While age, heart sizes (ventricular mass and EDV) and ventricular EFs varied considerably among the Fontan patients, the level of contractility was relatively narrow—between 50 and 80 kPa. These contractility values were comparable to the two biventricular controls, who had rest contractilities of 70 and 50 kPa, respectively. The values of relative tissue stiffness were consistent among *FPs#1-4, 6 and 7*, while they were higher in the remaining Fontan patients and both biventricular cases. The model-estimated resistance of the circulation—mainly given by the resistance *R*_*d*_ in our Windkessel model—were between 1.1 and $1.9\times 10^{8}\frac{Pa\cdot s}{m^{3}}$ for all *FPs*, and *R*_*d*_ correlated well with the vascular resistance assessed from data of patients (mean aortic pressure minus venous pressure divided by cardiac output), *R*^2^ = 0.851 (see Fig 7).

**Table 1:** Patients’ characteristics as directly obtained from the data at rest.

Patient	Age (years)	end-diastolic volume (mL)	EF %	ventricular mass (g)	preload (mmHg)	heart rate (bpm)	cardiac output (L/min)
*FP#1*	10	76	54	38	6.5	70	2.9
*FP#2*	7	102	45	49	5.6	62	2.9
*FP#3*	12	90	41	34	6.5	69	2.5
*FP#4*	4	58	41	27	5.9	82	2.0
*FP#5*	10	88	50	39	10.0	60	2.6
*FP#6*	17	95	51	52	6.7	59	2.8
*FP#7*	12	111	46	78	7.0	59	3.0
*FP#8*	6	55	65	51	9.2	69	2.5
*FP#9*	9	63	57	45	11.8	65	2.3
*CC#1*	1.5	20	63	20	11.0	103	1.2
*CC#2*	4	44	67	42	10.0	93	2.7

**Table 2:** Patients’ characteristics obtained by calibrating the patient-specific models at rest.

Patient	Proximal resistance *R*_*p*_ ($\times 10^{8}\frac{Pa\cdot s}{m^{3}}$)	Distal resistance *R*_*d*_ ($\times 10^{8}\frac{Pa\cdot s}{m^{3}}$)	Distal capacitance *C*_*d*_ ($\times 10^{-8}\frac{m^{3}}{Pa}$)	Venous capacitance *C*_*ve*_ ($\times 10^{-8}\frac{m^{3}}{Pa}$)	Relative tissue stiffness	Ventricular contractility (kPa)
*FP#1*	0.19	1.25	2.00	3.70	1.1	75
*FP#2*	0.17	1.10	1.80	2.60	1.0	70
*FP#3*	0.20	1.90	1.25	3.38	1.0	73
*FP#4*	0.43	1.70	1.00	1.60	1.0	80
*FP#5*	0.17	1.30	1.35	3.40	1.5	80
*FP#6*	0.18	1.30	1.15	1.67	1.2	56
*FP#7*	0.18	1.25	1.10	2.66	1.2	64
*FP#8*	0.31	0.93	0.70	1.12	1.4	50
*FP#9*	0.33	1.10	1.20	2.62	1.8	57
*CC#1*	0.78	2.10	0.32	0.67	1.7	50
*CC#2*	0.38	1.30	0.45	1.11	1.4	70

### Modeling outcomes during stress

Table 3 shows the increase of contractility, change of vascular resistance and of maximum achievable venous return $Q_{ve}^{max}$ in the model according to the data during stress. In all *FPs*, the contractility increased 2- to 2.5-fold. This was similar to the response in *CC#1*. In *CC#2* the contractility increased only 1.5-fold during stress.

**Table 3:** Summary of the changes in contractility, vascular resistance and venous return in the model for Fontan patients (*FP*) and control cases (*CC*) during dobutamine stress.

Patient	contractility at rest $\sigma_{0}^{rest}$ (kPa)	contractility at stress	distal resistance at rest $R_{d}^{rest}$ ($\times 10^{8}\frac{Pa\cdot s}{m^{3}}$)	*R*_*d*_ at stress	increase of max. venous return $Q_{ve}^{max}$ at stress (%)
*FP#1*	75	$2.25\times \sigma_{0}^{rest}$	1.25	$1.40\times R_{d}^{rest}$	10
*FP#2*	70	$2.50\times \sigma_{0}^{rest}$	1.10	$1.05\times R_{d}^{rest}$	50
*FP#3*	73	$2.25\times \sigma_{0}^{rest}$	1.90	$1.15\times R_{d}^{rest}$	45
*FP#4*	80	$2.25\times \sigma_{0}^{rest}$	1.70	$1.15\times R_{d}^{rest}$	45
*FP#5*	80	$2.25\times \sigma_{0}^{rest}$	1.30	$0.90\times R_{d}^{rest}$	20
*FP#6*	56	$2.40\times \sigma_{0}^{rest}$	1.30	$1.00\times R_{d}^{rest}$	50
*FP#7*	64	$2.25\times \sigma_{0}^{rest}$	1.25	$0.90\times R_{d}^{rest}$	45
*FP#8*	50	$2.00\times \sigma_{0}^{rest}$	0.93	$0.88\times R_{d}^{rest}$	0
*FP#9*	57	$2.50\times \sigma_{0}^{rest}$	1.10	$1.35\times R_{d}^{rest}$	-30
*CC#1*	50	$2.75\times \sigma_{0}^{rest}$	2.10	$0.88\times R_{d}^{rest}$	65
*CC#2*	70	$1.50\times \sigma_{0}^{rest}$	1.30	$0.90\times R_{d}^{rest}$	30

Fig 7 shows a good correlation (*R*^2^ = 0.842) between the myocardial contractility in the models and measured maximum ventricular pressure increase during isovolumic contraction, “max *dp*/*dt*”, a surrogate measure of myocardial contractility.

The reactivity of vascular resistance during stress is shown in Table 3. The resistance decreased by 10-12% in our two biventricular controls and in *FPs#5*, *7* and *8*. In *FPs#1, 3, 4* and *9*, on the contrary, the vascular resistance increased at stress, while it remained around the resting level in *FPs#2* and *6*.

The adaptions of the venous return law are shown in the upper left panels of Figs 5 and 6 for *FP#1* and *CC#1*, respectively. The corresponding maximum achievable venous return $Q_{ve}^{max}$ increase during stress is shown in Table 3 for all cases. The increase in *FPs#2-4, 6* and *7* was comparable to our biventricular control cases (30-65%), the increase in *FPs#1, 5* and *8* was however below 20%. Furthermore, the suggested level of maximum achievable venous return in *FP#9* even dropped by 30%.

## Discussion

In this study, we evaluated the added value of a biomechanical heart model [24] looped with venous return during diagnostic evaluation of patients with Fontan circulation with early-stage heart failure. We demonstrated that our proposed modeling technique improved the interpretation of XMR dobutamine stress exams and enabled patient-specific assessment of key components of the cardiovascular stress response.

### Model-derived properties at rest

As shown in Table 2, our model allowed to characterize the myocardial stiffness, contractile state and vascular resistance at rest in each patient. These quantities are not directly evident from the data, however, they can be relevant for clinical evaluation of Fontan failure. Moreover, they represent elements that may be clear potential therapeutic targets. For instance, patients with predominant issues with stiffness could be targeted with aldosterone antagonists; patients with contractile reserve limitation could be trialed on beta-blockers; and patients with vascular resistance issues could perhaps be managed with combination of angiotensin and neprilysin inhibition, to give some examples of a direct clinical significance and possible application of the modeling outcomes.

The myocardial contractility values in *FPs* were in the same range as these in our biventricular controls. The level of contractility in *FPs#1-5, 7* and in *CC#2* corresponded with the levels of active stress generated by sarcomeres of healthy hearts obtained during ex vivo experiments [33], suggesting that the contractile function in these Fontan patients was not significantly decreased at rest. This is an important gain, as the clinically used measure of systolic function—EF, which is load-dependent and therefore has a limited reliability—wrongly suggested an impaired systolic function in these patients with the EF values on or below the cut-off of normal ventricular function (Table 1). The contractility at rest in the remaining Fontan patients (*FPs#6, 8* and *9*) was lower and closer to the biventricular case *CC#1* (50 kPa). For *CC#1* this could potentially be a variation seen in age, as *CC#1* was considerably younger than the other patients, see Table 1. As the other Fontan patients did not show signs of heart failure at rest, the lower contractility value may not necessarily be pathological, especially when considering a good response of the contractility to the pharmacological stress discussed below. As age heterogeneity of the patients was large and the study population small, a conclusive comparison of the rest contractilites between the groups is impossible. Future studies with larger populations will shed more light on these differences.

The estimated myocardial passive stiffness values in *FPs#1-4, 6* and *7* shown in Table 2 correspond to the values that we are obtaining in healthy adult hearts (preliminary unpublished results, see study details [37]). This suggests that diastolic function was preserved and is keeping with our previous pressure-volume loop assessments in the same cohort which showed no relaxation abnormalities [38]. The biventricular subjects showed an increased stiffness in their systemic ventricles relatively to *FP#1-4*. This might be caused by their chronic and severe liver failure, which is previously shown to impact myocardial stiffness [39]. However, the difference in ventricular morphology (systemic left in the *CCs* vs. systemic right ventricle in the *FPs*) is also likely impacting these results. In *FPs#5, 8* and *9* the increased stiffness could be a key factor contributing to their early-stage heart failure. This is also suggested by the unusual high preload, which is detrimental for the passive flow through the lungs and increases systemic venous congestion in these patients. A detailed study of passive properties of the systemic left and right ventricle is out of scope of this article but will be addressed in our future work.

### Myocardial contractile reserve

Using the model adaptions during dobutamine stress, we were able to investigate the contractile response in each patient. We showed that all *FPs* had a good contractile reserve. The estimated increase of contractility by the factor of 2 to 2.50 is similar to the contractile reserve observed in *CC#1* and to the values reported in literature for healthy myocardium [40].

The model was also able to characterize abnormalities in the contractile responses to dobutamine stress, as shown by the detection of a blunted response in *CC#2*. Clinical notes revealed a hyperdynamic circulatory state in this patient with resting cardiac output (indexed to body surface area) being increased to 5.5 L/min/m^2^. This is a common feature in patients with Allagile’s syndrome and is a compensatory inotropic and chronotropic response to their liver failure, leading to exaggerated vasodilatation of the splanchnic and pulmonary vascular beds. The limited further increase of contractility (1.5-fold the resting value) seems therefore plausible.

We compared our model-obtained values of contractility to an invasive assessment of the surrogate contractility measure—ventricular max *dp*/*dt*—and showed that, both at rest and during dobutamine stress the model-obtained contractility values correlated well with the measurements (Fig 7). Of note, this correlation was achieved even though the ventricular size and morphology varied significantly among the patients. Additionally, the changes in contractility obtained using the model also correspond well with the changes in ventricular elastance previously obtained in our pressure-volume loop study [38]. While a direct measurement of contractility is unavailable *in vivo*, this good correlation throughout various physiological states and patients suggest that the model-obtained contractility estimates are valid. Some variation between the contractility estimates and max *dp*/*dt* values can be observed (see Fig 7 and Table 3). These differences are not unexpected: while the ventricular max *dp*/*dt* is only a surrogate measure of contractility, the model estimate is the actual measure of physiological properties at the sarcomere level. These results show that our model is able to estimate the myocardial contractility and contractile reserve in individual patients, which provides additional diagnostic information about the functional state of the ventricle.

### Reaction of vascular resistance to dobutamine

The model further allowed to quantify the effect of dobutamine on vascular resistance. Fig 7 showed that the model-estimated resistance *R*_*d*_ correlated well with the vascular resistance assessed from data of patients. This supports the role of *R*_*d*_ as the afterload of the systemic ventricle.

As can be appreciated in Table 3, the model-derived vascular resistance decreased at stress by 10-15% in our two biventricular controls and in *FPs#5, 7* and *8*, which is in line with pharmacological study [41]. In the remaining Fontan patients a counterintuitive increase of the resistance was seen during the stress. The non-physiological form of stress induced by dobutamine [42], in combination with the general anesthesia, are likely to significantly influence the vascular response observed in this study. However, the particular unusual increase of the vascular resistance in some patients might also be a component of their failure. Indeed, vascular disregulation has previously been reported and is regarded as a potential factor in the cascade of pathophysiological changes leading to Fontan failure [43].

While separating the response of systemic and pulmonary vascular resistance is not essential in estimating the contractile reserve, the role of the pulmonary vasculature and its reactivity to pulmonary vasodilation are of vital importance in investigating Fontan failure and optimizing patient management. The presented work is already introducing some key components to address these important clinical questions and further investigation of the vascular behavior, including separation of the systemic and pulmonary circulations, is a part of our ongoing work [44].

### Closed-loop heart-circulation modeling

We used a closed-loop model, in which the venous return determines the ventricular preload. The ventricular pressure record at rest (when HR is low) would allow us to directly prescribe the preload pressure according to the measurement and subsequently estimate the contractility at rest. Determining the preload pressure is, however, complicated at stress. A very fast HR limits the accuracy of the fluid-filled catheter for changes of low diastolic pressures. Moreover, prescribing the preload would restrain using the model only to interpret data and would not allow to investigate e.g. a mild decrease of heart rate (selective HR inhibition)—an example of *in silico* therapy investigation. The model with directly prescribed preload would therefore serve to augment the diagnostic value of combined exams, which is clinically relevant. However, a potential role of the model in predicting the optimal therapy in individual patients would be decreased. Even though this is not a scope of the presented work, it does represent a natural target for predictive modeling.

Our model of venous return is a simple representation which does not encompass all the complex interactions of venous return in neither the single nor biventricular circulation. However, it allowed the model to capture the observed behavior at stress while not directly imposing the preload, and therefore estimate the contractility at stress.

**Fig 8:**
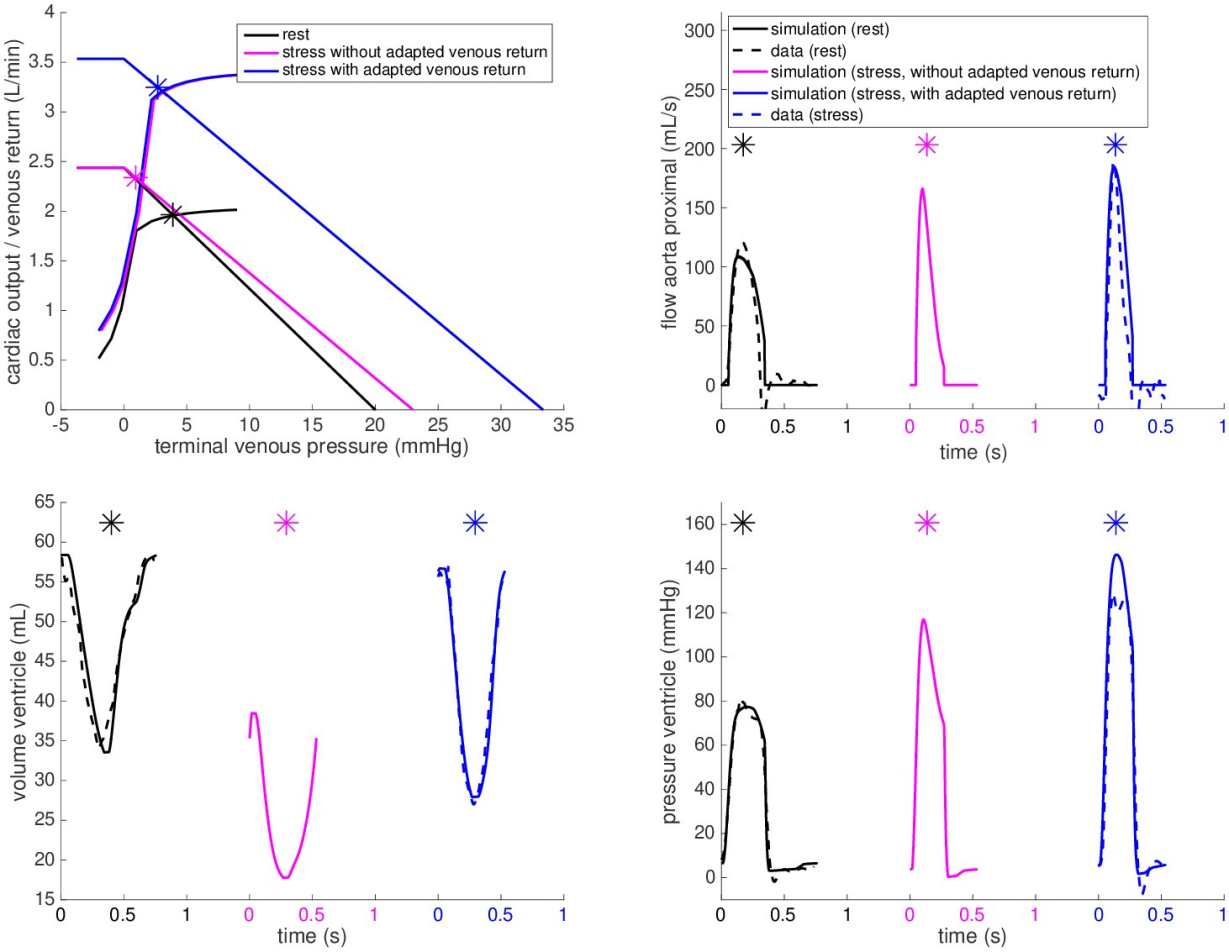
Effect of the venous return augmentation. Cardiac output curves of *FP#4* at rest (black) and during dobutamine stress with the cardiac output-venous return equilibrium points of the closed-loop system marked by asterisks—purple for the cardiac output at stress while the venous return kept as at rest, and blue allowing an increase of venous return ($Q_{ve}^{max}$ increased by 45%) during dobutamine stress.

In a healthy normally functioning cardiovascular system, the maximum achievable venous return flow $Q_{ve}^{max}$ is adapted during exercise, leading to a right and upward shift of the venous return linear relationship [28]. One of the contributors to this effect is the muscle pump—activation of the exercising muscles propagates venous flow towards the heart. The increase of *V*_*eff*_ in our venous return model causes such a parallel shift of the linear relationship and the increase of $Q_{ve}^{max}$. Fig 8 demonstrates on patient *FP#4* that if no modification of *V*_*eff*_ was introduced in the model of dobutamine stress (purple plots), EDV would fall to 38 mL, while the measured value was 57 mL. The calibration of *V*_*eff*_ (and hence $Q_{ve}^{max}$) allows a good match of the simulated vs. measured EDV at stress.

The changes in $Q_{ve}^{max}$ observed in our model are low compared to the values from literature for exercise stress. This is not surprising, given the non-physiological stress obtained during the XMR test—infusion of dobutamine during general anesthesia during which the effect of muscle pump is severely decreased. The missing components of the increased negative intrathoracic pressure (under physiological exercise due to deep inspiration) and the activity of the leg and abdominal muscles, which all help to draw blood to the chest cavity, can be addressed by exercise-stress CMR [45].

The model suggests a particularly unusual response of the venous system in *FPs#8-9*. In the latter, the maximum achievable venous return even decreased during stress. This might be artificial and caused by limitations of the modeling framework (particularly in the diastolic filling). However, the suggested capacity of venous return decreasing during stress could lead to more detailed investigations of venous return in such patients—targeted thanks to the model.

### Model-derived assessment of physiology

Using the calibrated closed-loop models during stress and evaluating the parameter changes from baseline, it is also possible to investigate the individual contributions of the components that make up the stress response. Patient *FP#4* has a patent fenestration—a shunt between the systemic venous system and common atrium that allows a portion of the systemic venous blood to bypass the pulmonary circulation and directly flow into the heart. An alternative view of Fig 8 allows to explore the model predictions of a hypothetical closure of the fenestration—which is known to lead to a better saturation of arterial blood by oxygen but could cause a decrease in ventricular filling (preload). The purple plots demonstrate the predicted limited venous return. As can be appreciated, this would lead to a lower preload and subsequently a decreased EDV, SV and CO. Even though this is only a hypothetical scenario (without having data for validation), these results illustrate how modeling could provide a better and personalized understanding of the potential impact of fenestration flow, collateral flow or other treatments aimed at improving flow towards the heart in Fontan patients.

**Fig 9:**
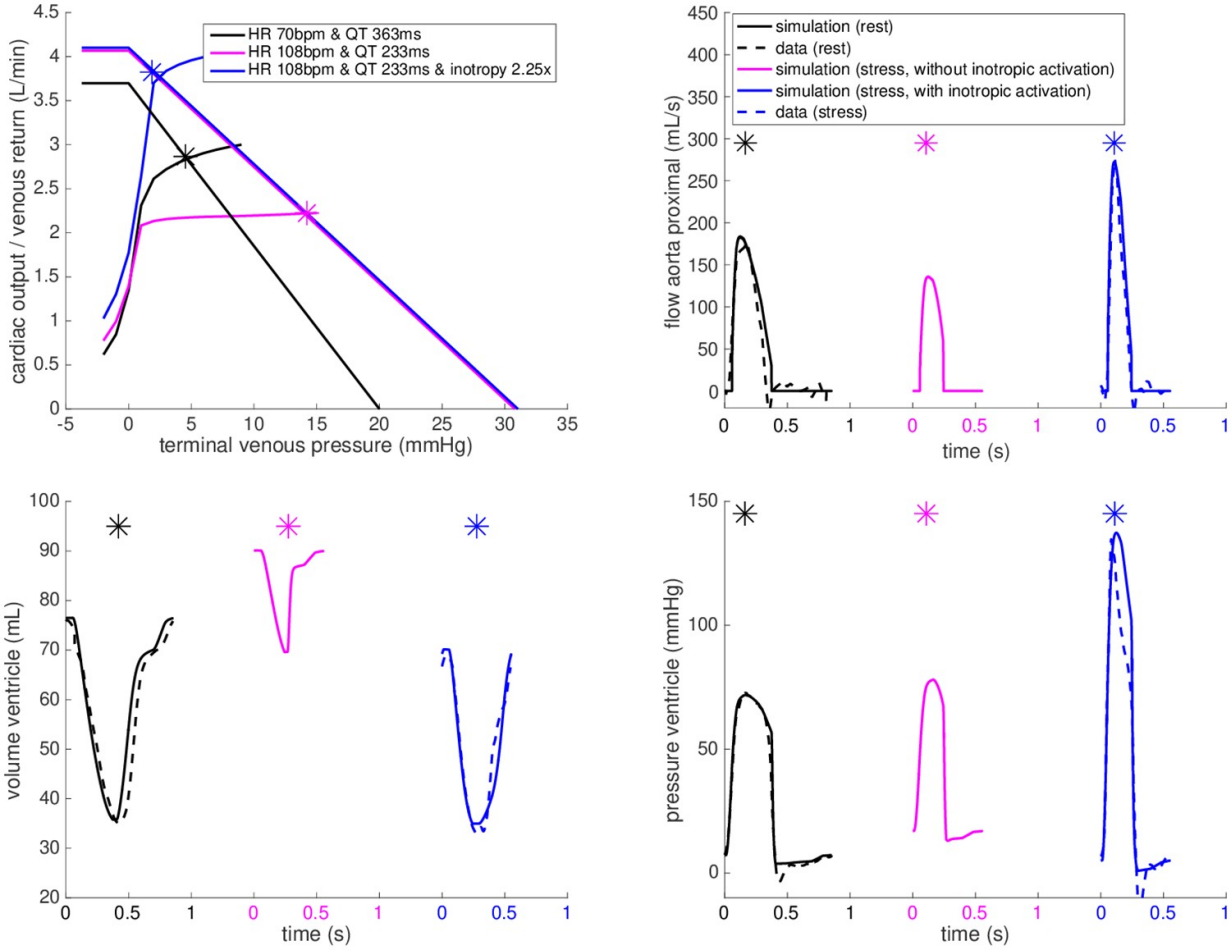
Chronotropic and inotropic effects. The closed-loop heart-circulation model for *FP#1* calibrated at rest (black), the predicted chronotropic effect for heart rate as during dobutamine stress but without the inotropic component (purple). The additional inotropic effect (blue) brings the simulation close to the data (dashed blue line).

Similarly, the purple plot in Fig 9 demonstrates the prediction of the model at stress in *FP#1* after adjusting the chronotropic effect (HR and ST segment duration) and adjusting the Windkessel and venous return models accordingly to the data, without including the inotropic effect. As can be appreciated from these plots, adjusting chronotropy alone would not lead to the increase in cardiac output (CO), systolic pressure and aortic flow observed during the exam. After applying the inotropic effect—by increasing the contractility in the model—the simulation reached the level of CO, ventricular pressure and aortic flow observed in the data (blue plots in Fig 9).

The limited complexity of our modeling framework allows medical doctors to set up and run patient-specific simulations by themselves, using variables directly relatable to known medical physiological principles. This opens the potential to directly include their clinical objectives and diagnostic challenges. We believe that in long-term this may contribute into an effective translation of such models towards clinical applications. As the simulations are run in 2-3 minutes, our model allows analysis of XMR data within a time frame that is compatible to the duration of clinical reporting of the XMR exams. Generating varying-elastance function directly by the patient-specific biophysical model [46] may be used to accelerate further the computation. Such a truly real-time approach could allow to augment directly during the XMR procedure the interpretation of results and possibly even to adjust the stress protocols “on the fly” in patient-specific way (e.g. no further increase of stress if the model-enhanced analysis does already provide a complete information for patient management). We however remark, that the “close-to-real time” approach, as presented in this paper, is sufficient when the aim is in augmenting the interpretation of already acquired data.

### Limitations

In this work, a simple linear venous return model was used to allow closed-loop simulations of the dobutamine stress response. The two points defining the linear venous return law—the maximum achievable venous return ($Q_{ve}^{max}$) and mean systemic filling pressure (MSFP)—are unknown and an average value of MSFP in Fontan circulations from literature was used in all subjects of our study. Table 4 shows a sensitivity study in two Fontan patients (one with low and second with high predicted increase of $Q_{ve}^{max}$) when varying the MSFP at rest between 15 and 25 mmHg—a reasonable range with respect to studies [35, 47]. Varying the assumed MSFP at rest within such a range did not change the estimate of contractility and contractile reserve, and the estimated $Q_{ve}^{max}$ changed only by less than 5% (an insignificant value, considering the increase of about 50% in biventricular cases). We remark that an estimate of this linear relationship might be obtained during invasive studies under general anesthesia by varying the level of positive end-respiratory pressure, and is subject of study in our future works.

**Table 4:** Sensitivity analysis for two selected Fontan patients when varying mean systemic filling pressure at rest (MSFP) between 15 and 25 mmHg: No change in the estimated contractility and contractile reserve, and the estimated change of maximum venous return varied by less than 5%.

Patient	MSFP at rest (mmHg)	Contractility at rest (kPa)	Factor of contractility increase at stress	Increase of max. venous return $Q_{ve}^{max}$ at stress
*FP#1*	15	75	2.25	5%
	20	75	2.25	10%
	25	75	2.25	10%
*FP#4*	15	80	2.25	45%
	20	80	2.25	45%
	25	80	2.25	45%

Patients with Fontan circulation are rare. This is reflected in the number of cases included in this study. However, Fontan failure represents a large burden in congenital cardiology due to challenges in diagnosing and treating these patients. In this proof-of-concept study, we coupled our model with the XMR stress exams. These exams provide very rich datasets, which enable the validation of our model, but are very invasive. Applying our modeling framework could potentially reduce the invasiveness of these examinations, for example by determining myocardial contractility using central systolic pressure alone. This can be obtained from peripheral measurements using e.g. transfer functions [48] or some other modeling techniques [49], and would eliminate the need of catheterizing the systemic ventricle.

A detailed sensitivity study for each of the parameters in the model is out of scope in this proof-of-concept study and we recall only to the sensitivity to the assumed MSFP in Table 4. In our future work we aim to include a larger cohort of patients to perform a detailed study of the sensitivity of our model predictions.

## Conclusion

We presented a biomechanical modeling framework for enhancing diagnostic assessment of pathophysiological exercise responses in patients with Fontan circulation, in whom conventional diagnostic assessment remains challenging. We showed that our reduced-order closed-loop heart-circulation model allowed the identification of key factors impacting Fontan hemodynamics—myocardial stiffness, contractility, contractile reserve and adaptations of vascular resistance—in individual patients. This enabled detailed investigation of the pathophysiological mechanism underlying early-stage heart failure in these patients and allowed the categorization of component analysis to give personalized therapeutic targets. With ongoing development and further proof-of-concept studies, our proposed modeling framework can provide a valuable addition to the current diagnostic assessment of Fontan patients.

## Supporting information

S1 Appendix Figures included in this appendix (S1 Fig 1-9) demonstrate the correspondence between simulations and data for the remaining patients included in the study. (PDF)

S1 File Data file *S1_file.txt* contains data in the form of time-vs- aortic and ventricular pressure, ventricular volume and aortic flow for all subjects as used in the study. (TXT)
