# Supplementary material for: Dobutamine stress testing in patients with Fontan circulation augmented by biomechanical modeling
Source: PLoS One. 2020 Feb 21;15(2):e0229015. doi: 10.1371/journal.pone.0229015 (PMC7034893; doi:10.1371/journal.pone.0229015)
Supplement: S1 Appendix — (PDF) [file pone.0229015.s001.pdf]

PLoS One Supporting Information S1 Appendix

Article title: Dobutamine stress testing in patients with Fontan circulation augmented by biomechanical modeling

Authors: Bram Ruijsink, Konrad Zugaj, James Wong, Kuberan Pushparajah, Tarique Hussain, Philippe Moireau, Reza Razavi, Dominique Chapelle, Radomír Chabiniok

S1 Figs 1-9 demonstrate the correspondence between simulations and data for the remaining patients included in the study.

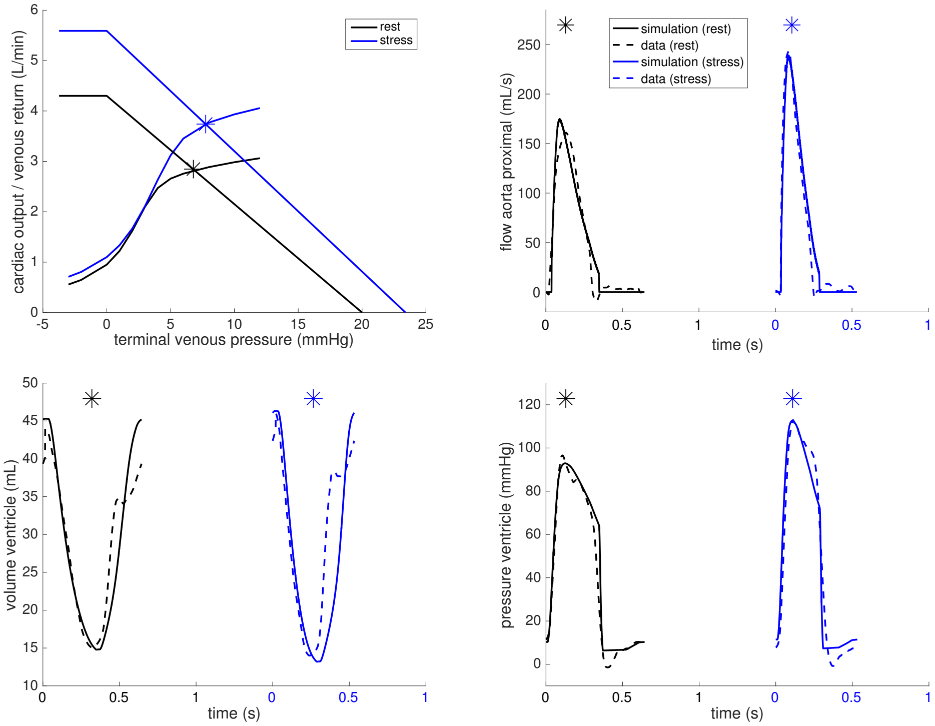

S1 Fig 1. Simulations for bivenricular control *CC#2*.

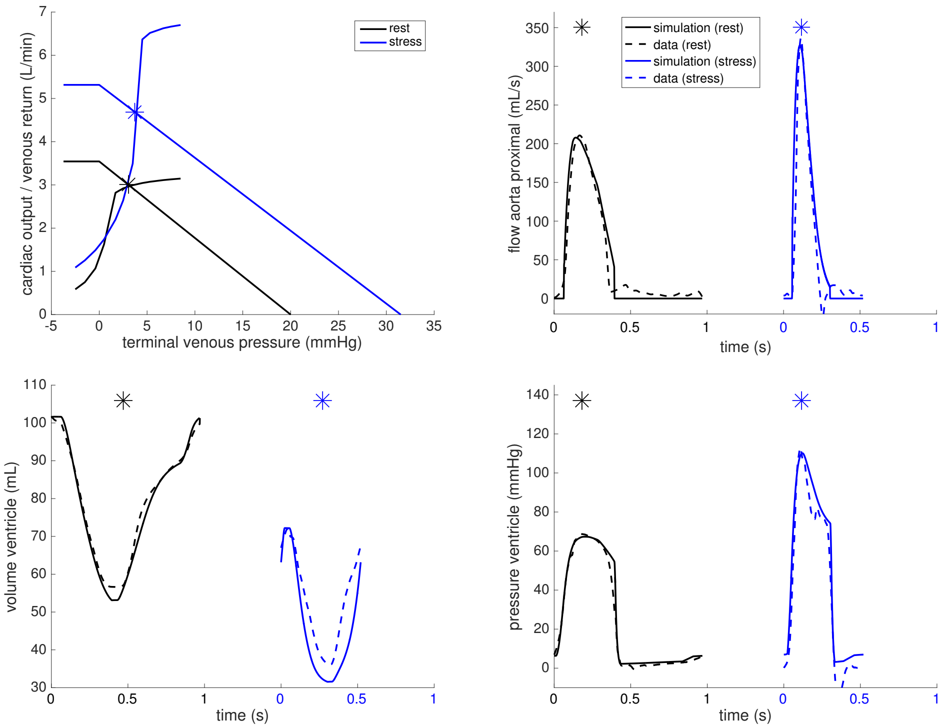

S1 Fig 2. Simulations for Fontan patient *FP#2*.

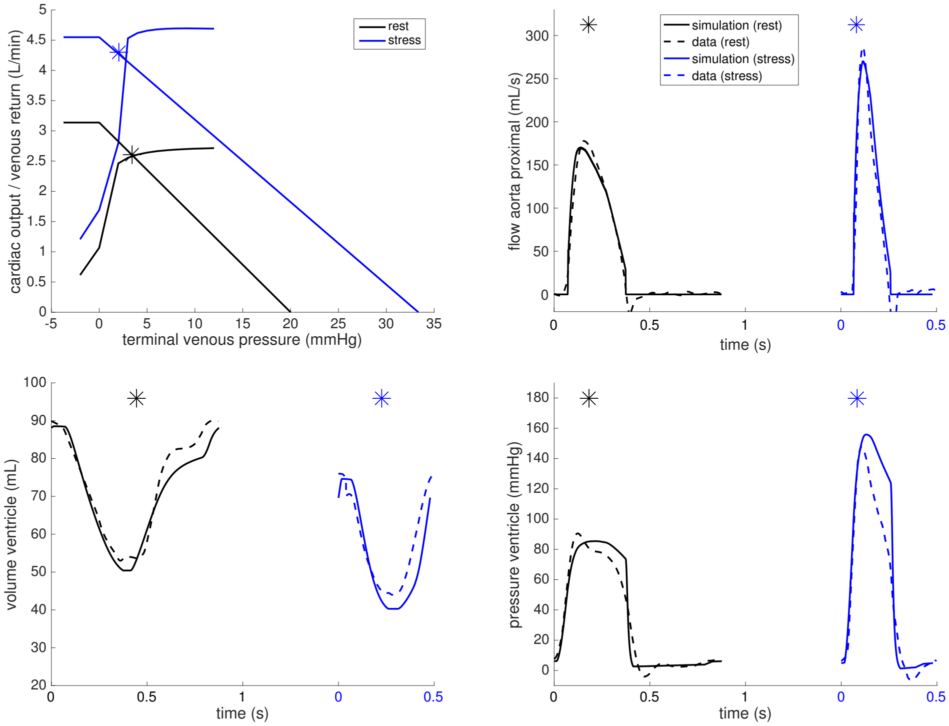

S1 Fig 3. Simulations and data for Fontan patient *FP#3*.

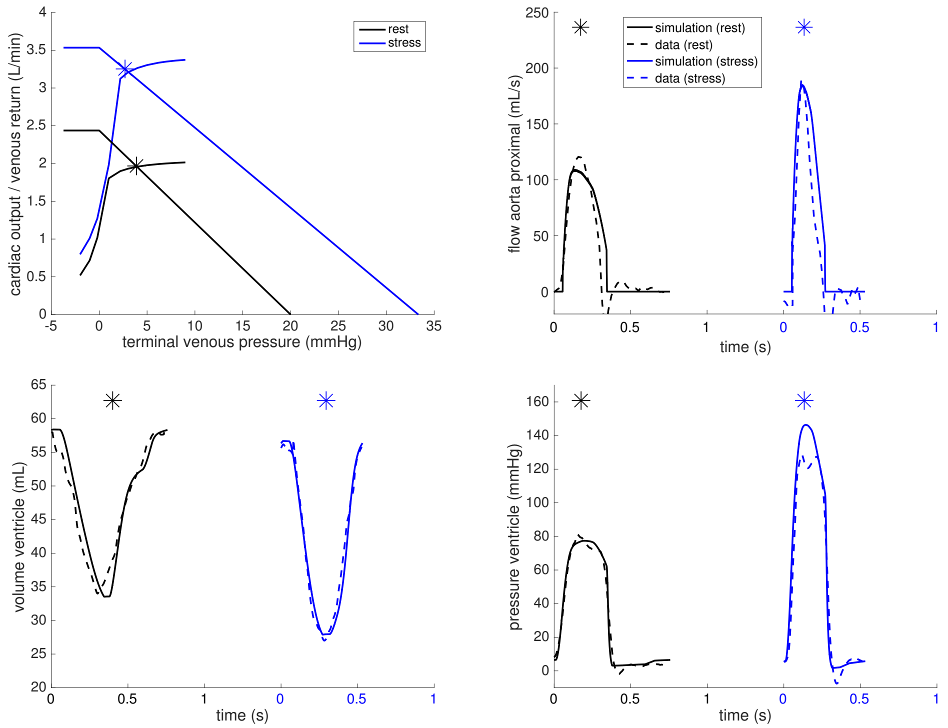

S1 Fig 4. Simulations and data for Fontan patient *FP#4*.

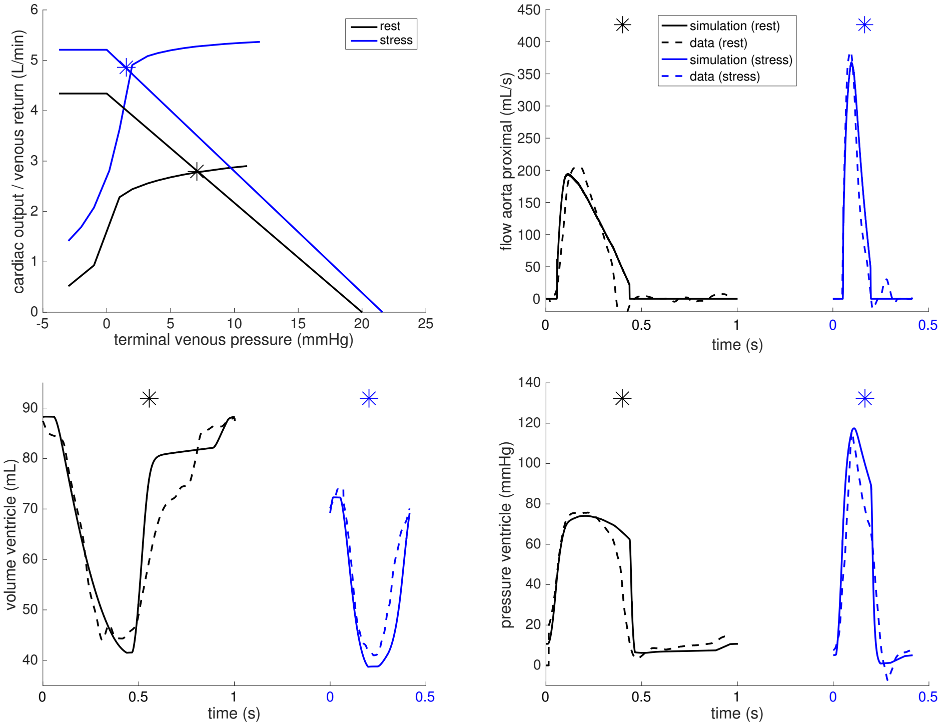

S1 Fig 5. Simulations and data for Fontan patient *FP#5*.

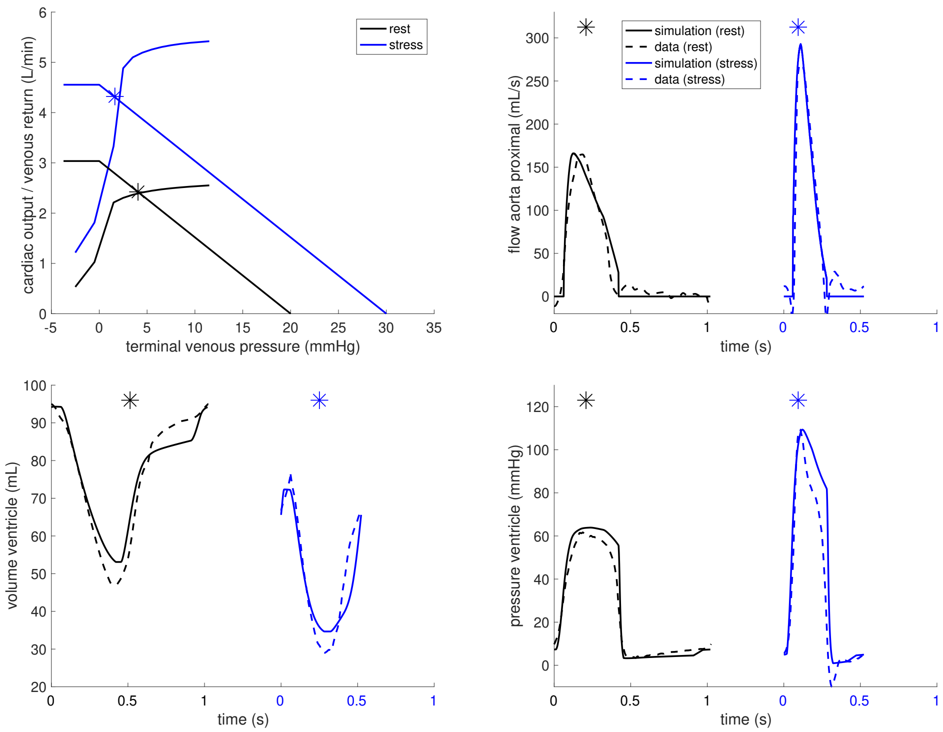

S1 Fig 6. Simulations and data for Fontan patient *FP#6*.

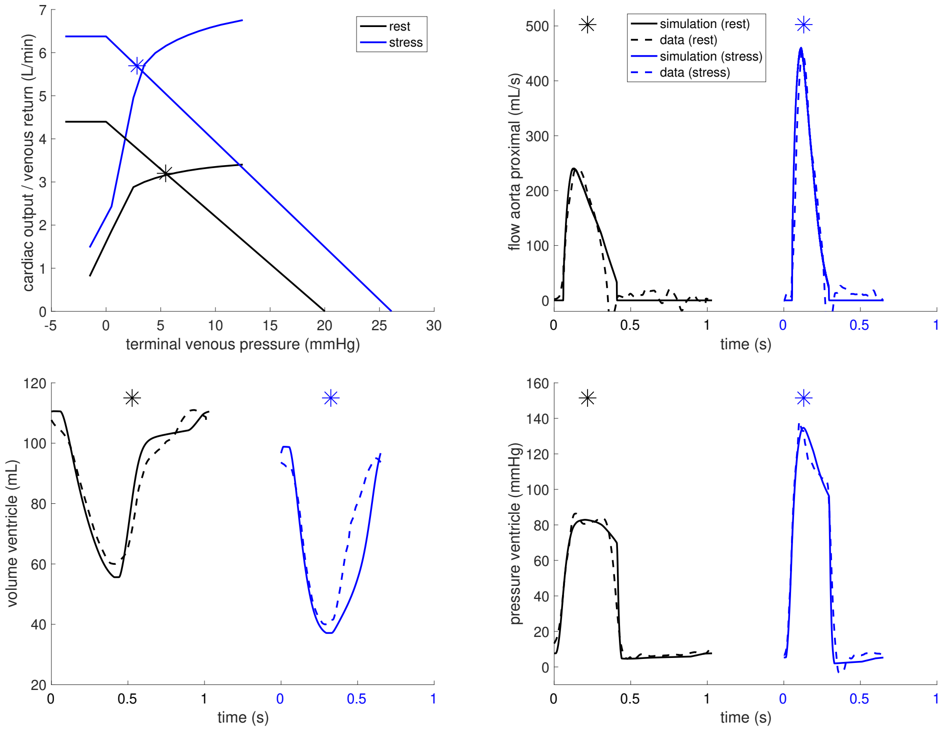

S1 Fig 7. Simulations and data for Fontan patient *FP#7*.

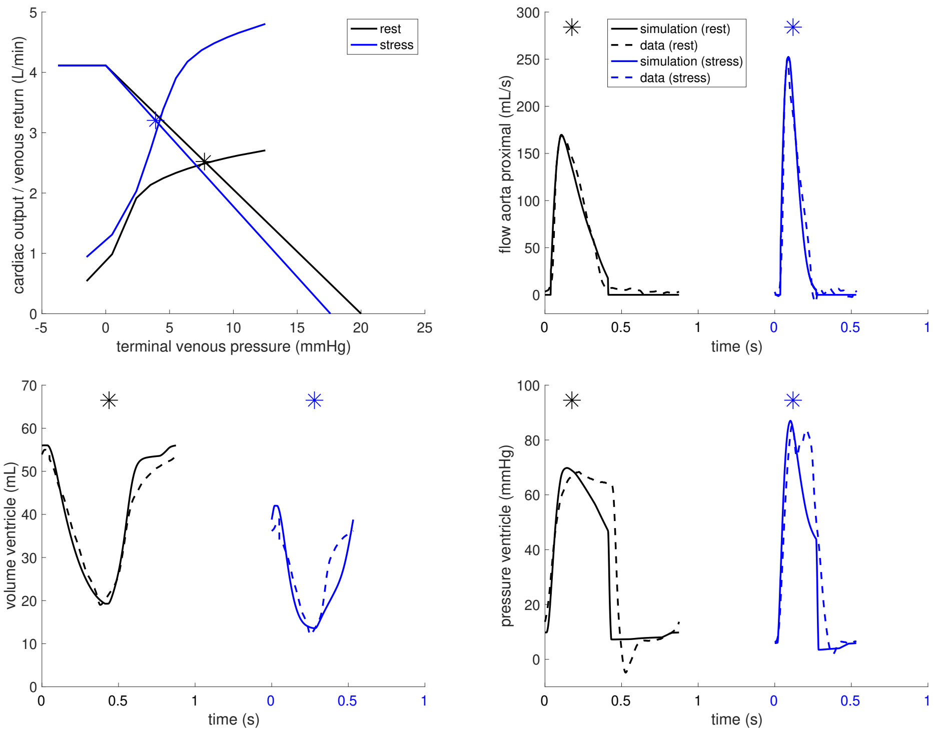

S1 Fig 8. Simulations and data for Fontan patient *FP#8*.

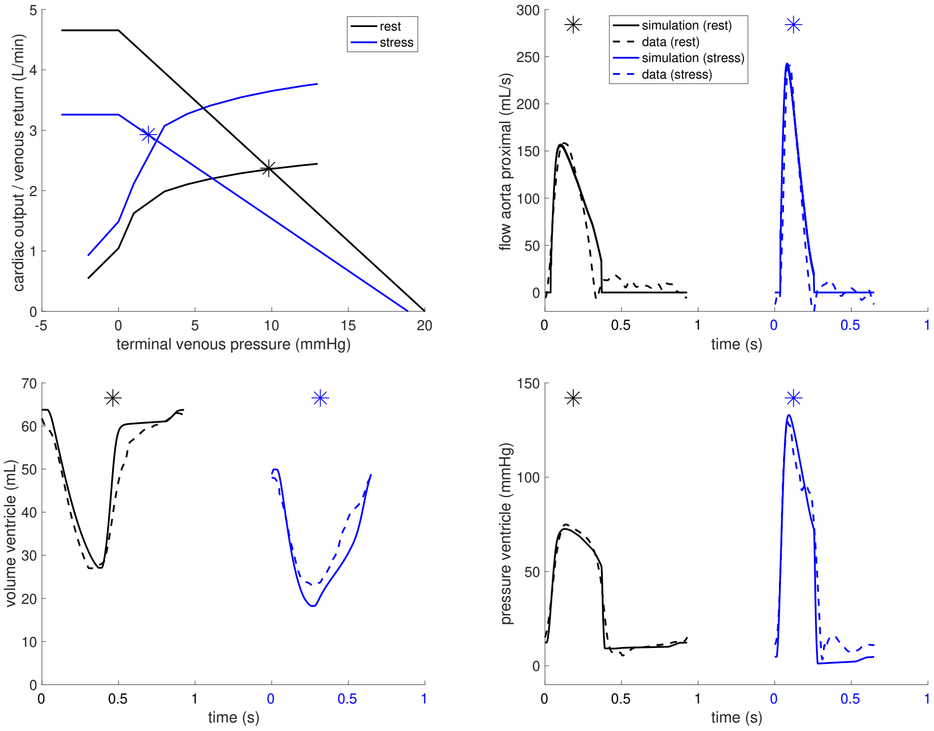

S1 Fig 9. Simulations and data for Fontan patient *FP#9*.
